# Critical slowing down may account for the robustness of development

**DOI:** 10.1073/pnas.2205630119

**Published:** 2022-06-22

**Authors:** Weiting Zhang, Pierluigi Scerbo, Bertrand Ducos, David Bensimon

**Affiliations:** ^a^Laboratoire de Physique de l’Ecole Normale Supérieure, Université Paris Sciences & Lettres, CNRS, 75005 Paris, France;; ^b^Institut de Biologie de l’École Normale Supérieure, Université Paris Sciences & Lettres, CNRS, 75005 Paris, France;; ^c^High Throughput qPCR Core Facility, École Normale Supérieure, Université Paris Sciences & Lettres, 75005 Paris, France;; ^d^Department of Chemistry and Biochemistry, University of California, Los Angeles, CA 90094

**Fig. 1.**
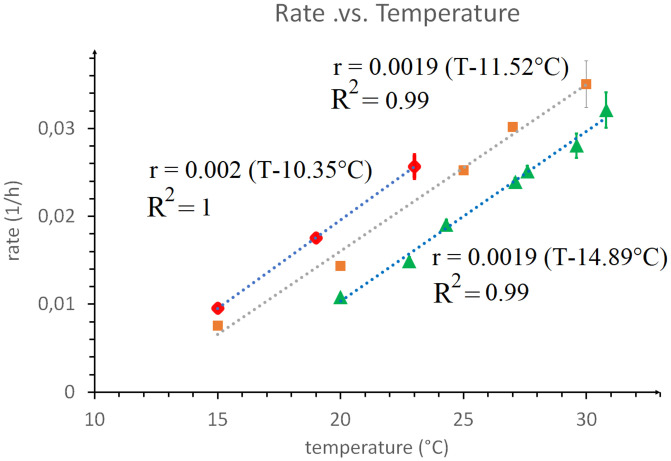
The developmental rate r of *C. elegans* (defined as the inverse of the developmental duration *T_S_*) as a function of temperature [red points are data from Filina et al. (2)] and a linear best fit (blue dots). Compare with the developmental rate of the fruit fly (*B. carambolae*) egg [brown points are data from Danjuma et al. (4)] and linear best fit (gray dots) and with the frequency (divided by 100) of the somitogenetic clock (in hours^−1^) in zebrafish as a function of temperature [green points are data from Schröter et al. (3)] and linear best fit (blue dots).

While the rate of development of poikilotherms can vary widely with temperature (and other external variables such as food availability, diet, etc.), the gene expression, morphology, and function of the organs of the embryo at all stages of development is independent of these external factors (1). In PNAS, Filina et al. (2) show that this is valid also in the development of *Caenorhabditis elegans* when the stage of development is defined relative to the overall developmental time, a property they call “temporal scaling.” We would like to point out here that this robustness could be a result of development occurring near a critical point, where all rates scale as *θ* − *θ_c_*, where *θ* is some external variable. Particularly, if development occurs near a critical temperature *T_c_* all developmental rates (growth rate, oscillatory frequencies, gene expression dynamics, etc.) should scale as *T* − *T_c_*. We have observed this to be valid during somitogenesis in zebrafish (1, 3). From the limited data in Filina et al. (2) it appears that this is also valid for the developmental rate of *C. elegans* (defined as the inverse of the development duration *T_S_*) (Fig. 1). Notice, though, that while “temporal scaling” is a consequence of criticality, the reverse is not true. The linear variation of developmental rates with temperature implied by the proximity to a critical point has also been observed in the development of the various larval stages in the fly (4, 5). It is thus tempting to hypothesize that a common mechanism is at work in these various organisms, possibly an essential transcription factor displaying critical behavior (1). If such a mechanism is shared by so distant organisms as *C. elegans*, fly, and zebrafish, it must be evolutionary very old, dating to the last bilaterian common ancestor appearing during the Cambrian explosion.
